# Barriers and opportunities to accessing social support in the transition from community to prison: a qualitative interview study with incarcerated individuals in Northern Norway

**DOI:** 10.1186/s40359-022-00895-5

**Published:** 2022-07-28

**Authors:** Line Elisabeth Solbakken, Rolf Wynn

**Affiliations:** 1 Department of Clinical Medicine, UiT The Arctic University of Norway, 9038 Tromsö, Norway; 2 Division of Mental Health and Substance Use, University Hospital of North Norway, Tromsö, Norway

**Keywords:** Prison, Incarcerated, Social support, Mental health, Recidivism

## Abstract

**Background:**

Incarcerated individuals have poor mental health compared to the general population. Social support has a beneficial effect on mental health. The buffering model proposes that social support facilitates coping under stressful conditions, while the main effects model suggests that belonging to social networks and having positive social interactions are beneficial for mental health. Prisons are a highly interesting context for studying social support, as imprisonment is perceived as stressful and disrupts social relationships and the availability of support. This study aims to explore incarcerated individuals' perceptions of social support from various sources in the transition from community to prison, its perceived significance for mental health, and the opportunities and barriers to accessing social support in a Norwegian prison context.

**Methods:**

The experiences of eight incarcerated individuals from a prison in Northern Norway were gathered through conducting individual in-depth interviews. The data analysis was inspired by Charmaz’s version of Grounded Theory.

**Results:**

Social support from peers was perceived to be important for the well-being and preserving of mental health in prison. Support from informal sources outside prison and prison officers were not granted the same significance by the participants. Although prison life was perceived as stressful, social support in the form of companionship, the feeling of belonging, shared activities, and everyday conversations were more important for the participants than support focusing on coping with the stress of incarceration.

**Conclusions:**

Peers are perceived to be the most important source of social support, and vital for well-being and mental health in prison. Barriers to support from family, friends and prison officers may amplify the significance of support from peers.

## Background

Health outcomes are affected by *the conditions in which people are born, live, work, and age* [1]. Some of the most central social determinants of mental health are social exclusion, adverse experiences in early life, poor educational attainment, unemployment, poverty, housing instability and poor access to health care [2]. People in prison generally experience challenges in all of these domains [3], and the social determinants for mental health and criminal behavior are largely overlapping [4]. The disadvantaged living conditions of people who end up in prison account for the immense burden of mental health disorders in prison populations globally [5–8]. The complex health and social needs of incarcerated individuals are an argument for providing health promoting prison environments [9], and gathering lay perspectives about health in a prison setting is pertinent [10]. This study explores incarcerated individuals’ perspectives on the disruption and re-establishment of social support in the transition to imprisonment, and the opportunities and barriers to social support in a prison context.

Social support can be defined as the perceived availability or actual provision of social resources in relationships [11]. Socially supportive actions include instrumental support (e.g., giving practical help), informational support (e.g., providing information, advice and problem-solving aid), and emotional support (e.g., listening and empathizing) [12, 13]. There is consistent evidence of a causal relationship between social support and mental health [14, 15]. Sufficient levels of social support are positively related to positive health outcomes and well-being [15–19], while low levels of social support are associated with physical and mental illness and even increased mortality risk [12, 20, 21 ]. Several studies have found that incarcerated individuals score significantly lower on measures of social support than people in the community [3, 22]. Among people in prison, a low level of social support is associated with mental illness and an increased risk for suicidal ideation and suicide attempts [23]. Studies have also found that higher levels of social support are associated with better in-prison adjustment and lower recidivism rates [24] and better mental health outcomes [25, 26]. Social support has an especially prominent role during circumstances perceived as stressful [12, 21]. Stress occurs when individuals appraise situational demands to exceed their coping capacity [27]. The well-established positive effect of social support on mental health can be explained by the *buffering model* and *main effect model* [28]. *The buffering model* proposes that social support moderates the effects of stress during crises and major transitions in life through facilitating coping strategies and adaptive behavior [29]. The *main effect model* suggests that integration in social networks provides positive experiences, a sense of stability and self-worth, promoting well-being in both stressful and normal circumstances [30].

The transition from community to prison is a period of intense stress for those who experience it [31, 32]. The despair accompanying this transition is reflected in the high rate of suicide in the first few weeks of imprisonment [33–35]. A literature review found that transitions that are unanticipated, involuntary, disruptive, negative and cause secondary problems are perceived as more stressful than transitions who hold the opposite characteristics [36]. Entering prison can be sudden and unexpected; it is certainly not voluntary; it is perceived by most as harmful and triggers an entire range of negative consequences like social stigma and loss of autonomy [31]. Adjusting to a new role as an incarcerated individual, finding one's place in the prison hierarchy, and adapting to institutional norms and the strict routines contribute to the stress of imprisonment [37, 38]. In addition, imprisonment disconnects individuals from their social networks and communities [24]. From the literature, we know that transitions that separate individuals from their established social networks, thus reducing the availability of social support, might heighten stress levels [36].

A significant source of informal social support for most people is close friends and family members. Prisons generally provide opportunities to maintain contact with friends and family on the outside through writing letters, making phone and video calls, and having visits. However, this contact is often severely restricted by institutional routines, time schedules, and security measures, and sparse contact with family members and friends on the outside is one of the most significant stressors for people in prison [38–40]. As support from family is associated with a reduction in reoffending rates after release [41], it could also have implications for public safety.

Incarcerated individuals' daily interactions are mostly restricted to other incarcerated individuals and prison staff, and their best chance of fulfilling social needs might be through forming relationships with their peers [42]. Several studies have found that naturally occurring support from other incarcerated individuals is regarded as important for mental health and well-being [26, 43, 44]. However, forming social affiliations with fellow incarcerated individuals also poses a risk. Trusting the wrong person can have profound negative consequences, like being subjugated to bullying or violence [42], and distrust in fellow incarcerated individuals is a prominent barrier to forming supportive social relationships in prison [38, 45].

Incarcerated individuals may also access support from formal sources like prison officers, priests, social workers, teachers, volunteer visitors, and health care personnel. Prison officers are the most central of these groups, as incarcerated individuals are dependent on them to fulfil their everyday needs. A study found that incarcerated individuals were willing to ask officers for practical help, while they seldom approached them for mental health issues and difficulty coping [46]. The work of prison officers in providing incarcerated individuals with information, listening to their concerns, and treating incarcerated individuals respectfully can promote mental health [47] and successful rehabilitation [48].

Social support profoundly affects mental health, and the stressful transition to prison severely disrupts contact with established social networks. Most of the previous research on social support among incarcerated individuals has focused on family networks’ role for successful reentry to society [49]. The role of social support during the transition to imprisonment and the reestablishment of social networks in prison have not been illuminated to the same degree. This study explores how the transition to imprisonment impacts the availability and utilization of different sources of social support, and the perceived opportunities and barriers to constructing new networks of support with fellow incarcerated individuals while incarcerated.

## Methods

### Imprisonment in Norway

Around 3000 people, of which approximately 23% are foreigners and 6% are women, are serving a sentence at any given time in one of Norway's 58 prisons [50]. In 2019, the average sentence length was 198 days [51]. The low incarceration rates [52], recidivism rates [53] and the humane prison conditions in Norway and the other Scandinavian countries have been denoted as “Scandinavian exceptionalism” [54]. The punishment is the deprivation of liberty, but the conditions in prison should otherwise approximate the conditions of society in general [55]. Seen from a non-Scandinavian perspective, prisons in Norway are small, uncrowded, and provide superior material comfort. Most prison wards have communal areas with a TV and a kitchen for cooking that allows for social contact between incarcerated individuals and visitation rooms that facilitate contact with family and friends [54]. Rehabilitation in the form of educational opportunities, work-related training, and treatment programs are available to incarcerated individuals [55].

### Ethics

The study design is based on the Helsinki declaration of medical research involving human subjects [56] and the Norwegian correctional systems' guidelines for research. All participants gave informed written consent. The study protocol was approved by the Data Protection Officer of the University Hospital of North Norway. The legal authority responsible for the welfare of the incarcerated individuals, i.e. the Norwegian Correctional System Region North also approved the study. Ethical approval was sought from The Regional Health Research Ethics Committee of Northern Norway, which deemed the project outside their mandate.

People in prison are considered vulnerable because of their loss of liberty and autonomy, poor health status, a high proportion of learning disabilities, and poor literacy skills [57]. Thus, it is necessary to take extra precautions to ensure ethical research conduct and the following method sections describe the measures taken in this study in further detail.

### Study design

This study adopted a qualitative inductive analysis approach inspired by Charmaz's version of Grounded Theory [58]. The data itself was the starting point for developing conceptual categories through iterative comparative analysis rather than analyzing data through the lens of preconceived theoretical categories. The study takes on a relativist ontological position that acknowledges that people perceive and interpret the world differently [59].

### Participants and study setting

Eight male incarcerated individuals participated in the study. Two were foreigners, and six were Norwegian, and the participants ranged in age from the early thirties to the late sixties, with a mean age of 48.3 years. The participants were held in a prison in Northern Norway with a capacity of around 60 persons, which is considered a medium-sized prison in a Norwegian context. Six incarcerated individuals were held in the high-security ward, and two were held in a low-security ward. The names of the participants presented in this paper are pseudonyms. Since there are few participants, it is necessary to withhold further details about their age, ethnicity, and sentences to preserve their privacy.

### Recruitment

The prison leader appointed a prison officer, the Reintegration Coordinator (RC), to assist the researcher in the interview process. Posters with an invitation to participate in the study were put up in the prison wards by the RC. The posters contained brief and easy-to-read information about the general aim of the study, that the interviews would be recorded, confidential, held at the prison healthcare ward and would last for approximately one hour. The posters encouraged those interested in participating in the study to contact the RC for more information. Incarcerated individuals who wanted to participate were then given more detailed written information about the study from the RC. This included information that participation was voluntary and about the right to withdraw consent at any time before, during, or after the interviews. It was specified that the information from the interviews would not be shared with correctional staff. In this study, all the incarcerated individuals who received additional information actively volunteered to participate by contacting the RC.

The RC scheduled the appointments between the incarcerated individuals and the first author, who conducted the interviews. Apart from receiving their first names and security level, the first author had no contact with or knowledge of the participants and their backgrounds prior to the interviews. All participants were given information about the study verbally from the first author and signed a written consent form prior to the interview. After the interview, the participants were encouraged to comment on the interview experience, ask questions and were reminded of their right to withdraw their consent.

The reimbursement of incarcerated people for participation in research has been debated [57]. Because of the relative deprivation of the prison environment, some argue that even small incentives could result in undue influence for participation in research. For this reason, we chose to abstain from providing incentives for participation in this study, to minimize the risk of undue influence.

### Interviews

Individual in-depth interviews were conducted with the participants in Norwegian. Since a substantial percentage of incarcerated individuals have learning disabilities and poor literacy skills, the information about the study, consent, and their rights as participants were thoroughly explained verbally before the interviews began. The interviews took place in an office in the prison's health ward and visitation rooms and lasted 60–90 min. The interviewer was alone with the participants and had a personal alarm connected to the guard room as a safety precaution. The interviews loosely followed an interview guide with open-ended questions about incarcerated individuals' knowledge of mental health, availability of mental health information, and where incarcerated individuals can find support and help if they are experiencing distress. The unstructured nature of the interviews allowed following up on the participants' individual experiences and perspectives. Social support was a common and spontaneously occurring theme among the participants and was considered essential for well-being and mental health. The interview guide was adjusted as a result of analysis of the first three interviews so that the questions served to refine and develop the categories related to social support for the subsequent interviews.

Recruiting people in prison for research participation is challenging due to ethical concerns and the need for facilitation from the correctional services. The shared prison context strongly influenced the perspectives of the participants, and the data set was sufficiently rich to identify common themes and contrasting perspectives. Thus, the authors judged a the relatively small sample size to be satisfactory to illuminate the research objective [60].

### Analysis

Each interview was transcribed in Norwegian by the first author within a week after its recording. The initial coding phase began after the first couple of interviews, and from there on, the analysis and collection of new data went in parallel. Interviews were included in the analysis as soon as they were transcribed, and the process of constant comparison was utilized to compare new instances to earlier instances in the search for patterns and contrasting perspectives. The coding was performed by the first author utilizing NVivo 12. In the analysis, transcripts were read line-by-line while systematically asking questions concerning the data. Coding in the initial phase encompassed labeling of segments of meaning ranging from a couple of sentences to small paragraphs. The most frequent and significant codes were utilized to organize data into conceptual categories in the next focused coding phase. The authors had several meetings to discuss the transcripts, tentative categories, and interpretations throughout the analytic process. Memos of ideas, analytical choices, and interpretations were used as a basis for this collaborative reflexive process. In the last analysis stage, the two authors interpreted and negotiated the results, and the final conceptual categories with representative quotes were developed. After completing the analysis, the quotes included in this report were translated from Norwegian to English.

## Results

### Imprisonment is a social transition

#### Disrupted relationships

Several of the participants described imprisonment as a considerable social transition, which entailed a reduction in social support from sources like friends and a fall in social status. John recounted how he went from being a busy and well-connected drug dealer to an experience of being disconnected from the outside world and socially irrelevant:

Then you go in here, and you lose everything. You do not feel important anymore. You've been walking around with three mobile phones that have been ringing 24/7, and then suddenly there are no calls. You are forgotten when you're in here. No-one contacts you. No-one sends you letters. **John**

For some, the transition was abrupt with no opportunity to make plans or say goodbye to loved ones, for example in instances where people were put on remand during the investigation of their (alleged) crimes. One of the other participants told the story of his time on remand with solitary confinement:

I was pulled out of society, from my family, from my freedom and everything, and was put into a room where I had just one hour a day alone in a small yard with four walls and no roof. It was tough (…). I was unsure if I was going to make it or if I should just give up. **Fred**

This incarcerated individual could not contact his family and friends for over a month, and during this time his only human contact was with prison officers and health care personnel. People on remand with solitary confinement do not participate in work or other activities, and the isolation was so hard to handle that Fred questioned if he would make it through.

The experience of being socially isolated was a common theme among most of the participants. Like John and Fred, the other participants also shared stories of being cut off from their social network. The people that used to be closest to them, the ones that they would turn to for support and help, were no longer available to the same degree as before. The participants talked of barriers for keeping in touch with friends and family like security measures, endless bureaucracy when applying for and leaving on furlough, limited phone hours and the expense of calling when you have scarce resources.

Often you can't get in touch with them. When you call people, right, and the caller-id is "unknown". Many people do not answer calls with unknown caller, they think it is telephone marketing and stuff. It is such a disappointment, and then you must write letters, which takes like forever. **David**

Several of the incarcerated individuals also expressed a need to confide in their family members and tell them how they were really doing, but that they were unable to because the phone calls were monitored for security reasons:

You do not risk calling from a phone in here, to spill your guts, because the phone is monitored. They say it isn’t down here [lower security level], but no-one believes it. **John**

The inmates feared that the things they talked about on the phone with their friends and family would have consequences for them later on:

You have to consider carefully what you are saying. Everything is recorded. And everything is… It is used against you later. **Kenneth**

#### The perceived role of peer support for mental health

Imprisonment disrupts relationships with friends and family. Simultaneously, it provides the opportunity to form new social connections and networks. In the interviews, the participants were asked how one could take care of one's mental health in prison. They were unison in their answer that forming relationships with other incarcerated individuals is one of the most important things you could do:

They [new incarcerated individuals] would have to find some friends they can trust. Someone that they can talk to and be with. To get information from and learn how things work around here, and to ask for advice. **Paul**

Several of the participants underscored the need for information as a new incarcerated individual, as life within the prison walls was fundamentally different from life on the outside:

Someone you can talk to… …it doesn't have to be a lot. At least when you're in prison, because then you are bare naked. You don't know anyone. You have nothing. So, if you find someone to talk to, that can give you some answers, right. … It's a gift from above. You have no idea. **Kenneth**

The first-time incarcerated that come here are very withdrawn. And I observe how they are slowly but steadily breaking down. And I try to talk to them. I say that they must not take it so seriously, that they have to calm down. They should smile and be nice to the officers and try to forget about all of this. **Gary**

Gary explained how he attempts to help new incarcerated individuals by telling them that their convictions are not the end of the world, and that they should try to go on with their lives. He also advised them on how to behave towards the prison officers, which can be considered essential advice for adjustment to prison life.

Several of the participants expressed concern for the well-being of people who self-isolate from the community of incarcerated individuals. Some of the participants explicitly linked self-isolation to mental health problems, and they expressed the belief that increasing social contact would be beneficial for those who are isolated:

And there are people that you cannot get in touch with here. They are bitter and isolated. They do not interact with others during the little time we have in the shared living room on the ward. It is them against the clock. They're killing time. They have a [release] date, and that's it. **Roger**

I try to lure them out, and I'm trying to talk to them and engage them. Because I know it's tough. You can see it in their wrinkles [makes a frown]. **David**

However, the participants did not share the same concern for a particular sub-group of incarcerated individuals. Individuals convicted of sexual offences have a low status within the prison walls, and several of the participants described a rather intense antipathy. Here exemplified by Kenneth’s story of how the officers threatened him and his prison wing mates that they would be moved if they did not socialize with a person convicted of sexual offences:

You are not f…. going decide who I'm talking to in here. That's up to me!" And then they decided to move him instead (…) I cannot! I can't! I can't see, and act, and be… First of all, I've got nothing to talk to such people about! **Kenneth**

### Prerequisites for social support in a prison context

#### Talk and trust in a community of criminals

Although most of the participants acknowledged the need for forming relationships with other incarcerated individuals as important for mental health, they were also ambivalent about it for different reasons. Knowing who you could trust, and how much you could tell them was an issue that had to be considered carefully:

Okay, I am not going to say more than I can accept could come out to other people (…) If you say something to one person in here, the community is so small, and then he's got a friend that he tells it to, and then he's got a friend, and so it goes. It's like the Chinese whisper game from when you were a child. **John**

Seeking emotional support from other incarcerated individuals could pose a risk to their social status. David seemed to believe that revealing to others that he was having a hard time in prison would damage his image as a “tough guy”. He kept his worries to himself, even though he believed that his fellow incarcerated individuals were also having a hard time:

You'd feel whiney, and you've got a rough image. And it's not always okay to talk about these things. And you are alone with it, and your mind is churning. And I think there are many that… They walk around like this [bends his neck]. And they're trying to keep their head above water, like me. **David**

Another aspect that some of the incarcerated individuals pointed out was that they did not see the relationships they formed in prison as lasting beyond their sentences, and this impeded their willingness to trust and confide in their fellow incarcerated individuals:

Some things are private. You do not want to share them with everyone. Not an incarcerated person that you know will only be doing a year or two. You do not know what kind of person they are. **Michael**

Despite trust issues, several of the participants expressed a strong need to have conversations with others, but said that they found it challenging to connect with other incarcerated individuals because the conversation topics in their prison wing often revolved around criminal activities:

It is insane that they [fellow incarcerated individuals] are sitting at the prison wing talking about drugs, and about violence, and about… It makes your flesh crawl **Roger**

#### Shared activities, space and time

Many of the participants highlighted shared time, space and activities as important factors in forming relationships with other incarcerated individuals:

Since we live in close quarters, we quickly get know one another (…). And like, you find, uh. Someone you get along with and who you can talk to. **Fred**

I have three friends. We play cards. We make meals together. They save me and keep me on my feet. **Paul**

In prison, time spent in communal areas, in the prison yard, and in joint activities provides opportunities to form and maintain social relationships with other incarcerated individuals. Most of the participants said that they wanted more organized activities with other incarcerated individuals, like sports and peer-support groups.

#### Institutional restrictions

All the participants spoke of how their everyday lives were strictly governed by the prison rules and routines:

The structure in it all. And these rules. The day is set. Everything at set times, and so it goes. There’s no room for flexibility. It’s SO hard. **David**

Since incarcerated individuals have little influence over their everyday lives, and their movement and communications are restricted, social support is not necessarily available for incarcerated individuals when they need it. Several of the participants who had had earlier convictions claimed that reductions in budgets for the Norwegian correctional service in later years had led to fewer activities and more time locked up in their cells:

And what happens is that you are alone much of the time. You are locked in your cell. **Kenneth**

It is a storage box. There are very few activities available compared to other prisons. **David**

This, of course, had consequences for the time spent with other incarcerated individuals, and for the opportunities for accessing social support. One of the incarcerated individuals said he even took on chores to keep himself busy and to have social contact with others:

I ask them if they can lock me out so I can help clean the toilets and do other stuff. Then you can have eye contact with others, and we talk a bit and can have a cup of coffee together. **Roger**

### Perceptions of support from prison officers

#### Unequal access to support

Prison officers represented a variable and uncertain source of support for the participants, as only some of the prison officers were perceived as helpful and that they most of the time had to rely on other incarcerated individuals to get the information and practical help they needed:

Honestly. Straight from my heart. The help you get here equals zero. Incarcerated people must help each other. **Paul**

They do not help you with anything. They don't tell you how things work in here. You have to ask to get to know. And obviously, if you have mental health problems before you come in here, then you might not dare to ask, and soon you'd be lying there rotting in your cell. **Kenneth**

Several of the incarcerated individuals shared positive stories about receiving support and help from some of the prison officers, but they all underscored that they had to make deliberate decisions about whom they asked for assistance:

We have some officers that care. They actually ask: How are you today? Just tell me if there's something you need to talk about. You can talk to the officers. At least the ones you get along with. You do not have a good relationship with everyone. **Fred**

Fred was among the participants with the most positive attitude to prison officers, and the rehabilitative aspects of prison. He said that he wanted to make the most of his time in prison by building new skills through schools and activities and staying positive. Gary, on the other hand, opposed the whole idea of both the punishment and the rehabilitative aspect of prisons. He believed that most of the incarcerated individuals were in a worse state after finishing their sentences. Gary found it difficult to get the help he needed from the officers:

It's not possible to have a normal human conversation, one-to-one, with a correctional officer, saying that: "I like, need someone to talk to now, and…" "No, we don't have the time". Right? So I have asked them a lot of times now: Why-Why are you even here? If it hadn't been for us, you like, wouldn't have a job. What are you doing here, really?" **Gary**

#### The power imbalance: a barrier for support

The power imbalance between prison officers and incarcerated individuals is a significant barrier for seeking support. Michael tells a story of how he requested access to his electronic records. To his surprise, the mental health issues he talked about with his primary contact officers were written in the prisons’ electronic records and information he regarded as private was available to other prison officers:

You talk to a person, and you think you're only talking to that person. But then you read it [the records], and then you are aware than maybe someone else can access and read it to. **Michael**

Other participants also expressed concern about the prison records and gave this as a reason for not confiding in officers about personal matters. Several of the participants feared the officers would use personal information against them:

Some of the prison officers you can talk to about this stuff. But it is very limited when it comes to these kinds of things, how many prison officers you are willing to confide in. Because some of them will use it against you afterwards. You get very suspicious in here. **John**

Although most of the participants told stories about prison officers that had helped them in one way or another, they were ambivalent at best, but mostly critical of the perceived lack of support they experienced from prison officers.

## Discussion

This qualitative interview study explores the role of social support in the transition from community to prison and the re-establishment of social networks within the prison walls. The participants' perspectives in this study also shed light on how a prison context may influence the accessibility, acceptability, and utilization of different sources and types of social support. In accordance with some prior research [38, 40], we found that participants experienced severe distress from the transition from community to prison. Most of the participants felt isolated and confused as they were initially left to deal with the shock of imprisonment on their own. The participants underscored the importance of forming social relationships on the inside, and perceived support from peers to be more accessible and acceptable than support from other sources.

Informational support that facilitates understanding of situational demands, and advice on effective coping strategies are essential in stressful situations [12]. Several of the participants claimed that advice and information from fellow incarcerated individuals were crucial for coping with the adjustment to prison life. This finding aligns with the *stress-buffering model* of social support. However, other types of coping-focused support, for instance, emotional support, appeared to be less common. Some participants expressed concern about being perceived as weak if they talked to fellow incarcerated individuals about their problems and were cautious about trusting their peers. A probable explanation for this is that showing weakness or revealing personal information to the wrong person could make them vulnerable to exploitation or bullying from other incarcerated individuals [42]. Most of the participants highlighted the feeling of belonging and normal everyday conversations and activities with other incarcerated individuals as essential for their well-being and mental health. This finding corresponds to the *main effects model* of social support [21], emphasizing the positive effects of everyday interactions and belonging to social networks [26].

We found several conditions that shaped the perceived acceptability and availability of support from prison officers. The perception that many officers were unwilling to be of service, a perceived lack of confidentiality, and the fear of officers using personal information against them, were barriers to seeking support from prison officers. These findings elaborate on earlier research that suggests that incarcerated individuals are reluctant to seek emotional support from prison officers [3, 46]. From other studies, we know that incarcerated individuals perceive the relationship with prison officers as important to their well-being [26, 47].

Facilitating contact between incarcerated individuals and their families is particularly important since it is consistently associated with better rehabilitative outcomes and a reduction in reoffending after release [41]. The participants in this study experienced significant barriers to contact with their friends and family on the outside. From the perspective of a correctional service, contact with people from the outside constitutes security risks such as the potential for criminal planning and smuggling of unauthorized items [61]. Thus, surveillance is necessary for both prison safety and public safety. The findings in this study suggest that the security aspects of the correctional service are barriers to social support from people outside prison. Interestingly, the asymmetrical power relationship between incarcerated individuals and officers also affected the availability of social support from friends and family on the outside. For instance, several of the participants perceived the monitoring of phone calls as a barrier to talking about subjects of a more emotional and private character.

Based on the present data, we propose that the barriers to support from friends, family, and prison officers amplify the significance of support from the more accessible fellow incarcerated individuals. However, the participants also described barriers to social contact with their peers. Security measures and institutional routines strongly govern how, when, and where social interaction can occur, as incarcerated individuals eat, sleep, work, and spend their leisure time according to prison routines. Other studies show that shared space and time are essential for forming supportive relationships [43, 45]. The participants in this study experienced that social support from fellow incarcerated individuals was obstructed by the participants being locked up in their cells much of the time, few shared activities and by self-isolation. Several of the participants pointed out that the pruning of the budget for the Norwegian Correctional Service in later years has led to more time locked up in the cell and a reduction in activities. This claim is supported by the annual report from the Norwegian correctional service that maintains lower funding has resulted in a cutback in activities, in-prison rehabilitation programs and, a significant reduction in prison staff in the Norwegian correctional system [62]. A survey from 2018 by The Norwegian Directorate of the Correctional Service showed that one-third of incarcerated individuals spent more than 16 h a day locked up in their cells [63]. This indicates that considerable proportion of Norwegian incarcerated individuals spends less time in meaningful activities than the recommended minimum of eight hours a day [64].

In this study, several prominent barriers to social support are related to institutional and public security. Reducing the barriers directly associated with security could compromise the safety of incarcerated individuals, correctional staff, and society in general. Thus, balancing the need for rehabilitation against public safety is a challenge. Some studies have found that contact with criminal peers in prison can reinforce criminal behavior and increase the risk of recidivism [65, 66]. In this study, several participants mentioned criminal talk in the ward as a barrier to experiencing meaningful social interaction with peers. We believe this is one argument for increasing the amount of organized social activities in prisons. While activities in prison may increase opportunities for accessing social support, they could also decrease criminal influence as interaction with peers may become more centered on organized social activities. In addition, shared activities have the potential to promote positive staff-incarcerated individual relationships.

Correctional services can also increase social support by facilitating the organization of peer-based interventions where incarcerated individuals provide advice and support to fellow incarcerated individuals. A systematic review indicates that peer listening services that offer advice and emotional support have positive effects on well-being, coping, and rehabilitation of both receivers and providers [67]. The results from our study indicate that organized peer support could be especially important during the first few weeks in prison. In this study, the participants' accounts also reveal sub-groups of incarcerated individuals that could be at increased risk for having insufficient access to social support, those who self-isolate, and individuals convicted of sexual offences. Although we lack direct information from individuals convicted of sexual offences in this study, we know that they are more likely to be socially isolated than other incarcerated individuals [31, 68, 69]. Thus, peer-based support interventions could be especially important for this group.

The results of this study contribute to a greater understanding of the contextual determinants for social support. The prison conditions in Norway and the other Scandinavian countries are known to be humane compared to other parts of the world [54]. Nonetheless, we found significant barriers to accessing support from both informal and formal sources, which had perceived consequences on the well-being of the participants. Since most incarcerated individuals return to their communities, the mental health of this underprivileged group is important to public health [70]. A complex interplay of biological factors and social determinants shapes the health and criminal paths of those who end up in prison. Most incarcerated individuals have a history of poor mental health before imprisonment [66], and the most prominent causes of mental illness are not rooted in the conditions of the correctional service. However, these pre-prison vulnerabilities make it even more critical that correctional services aim to preserve and promote the mental health of incarcerated individuals.

## Limitations

The data in this study is based on interviews with only eight self-selected participants from just one prison. We do not assume that the results necessarily represent the Norwegian correctional setting. However, we believe we have provided sufficient information about the participants, method, data, and context for others to judge the transferability of the results to other correctional contexts. We hope that our findings may encourage more research on the vital role of social support for the well-being of people in prison.

## Conclusions

Peers are perceived to be the most significant source of social support for the participants. Shared activities, conversations, and companionship are experienced as vital for well-being and mental health in prison. Barriers to support from friends and family and prison officers may amplify the significance of support from peers.

## Data Availability

The datasets generated during and analyzed during the current study are not publicly available due to privacy concerns, but are available from the corresponding author on reasonable request.
